# Antiepileptogenic Effects of Anakinra, Lamotrigine and Their Combination in a Lithium–Pilocarpine Model of Temporal Lobe Epilepsy in Rats

**DOI:** 10.3390/ijms242015400

**Published:** 2023-10-20

**Authors:** Olga E. Zubareva, Denis S. Sinyak, Alisa D. Kalita, Alexandra V. Griflyuk, Georgy P. Diespirov, Tatiana Y. Postnikova, Aleksey V. Zaitsev

**Affiliations:** Sechenov Institute of Evolutionary Physiology and Biochemistry, Russian Academy of Sciences, 194223 Saint Petersburg, Russia; zubarevaoe@mail.ru (O.E.Z.); den67920405@yandex.ru (D.S.S.); st076008@student.spbu.ru (A.D.K.); griflyuk.al@mail.ru (A.V.G.); diespirov.gp@yandex.ru (G.P.D.); tapost2@mail.ru (T.Y.P.)

**Keywords:** temporal lobe epilepsy, anakinra, lithium–pilocarpine model, behavior, epileptogenesis, hippocampus, spontaneous recurrent seizures, neuronal loss

## Abstract

Temporal lobe epilepsy is a common, chronic disorder with spontaneous seizures that is often refractory to drug therapy. A potential cause of temporal lobe epilepsy is primary brain injury, making prevention of epileptogenesis after the initial event an optimal method of treatment. Despite this, no preventive therapy for epilepsy is currently available. The purpose of this study was to evaluate the effects of anakinra, lamotrigine, and their combination on epileptogenesis using the rat lithium-pilocarpine model of temporal lobe epilepsy. The study showed that there was no significant difference in the number and duration of seizures between treated and untreated animals. However, the severity of seizures was significantly reduced after treatment. Anakinra and lamotrigine, alone or in combination, significantly reduced neuronal loss in the CA1 hippocampus compared to the control group. However, the drugs administered alone were found to be more effective in preventing neuron loss in the hippocampal CA3 field compared to combination treatment. The treatment alleviated the impairments in activity level, exploratory behavior, and anxiety but had a relatively weak effect on TLE-induced impairments in social behavior and memory. The efficacy of the combination treatment did not differ from that of anakinra and lamotrigine monotherapy. These findings suggest that anakinra and lamotrigine, either alone or in combination, may be clinically useful in preventing the development of histopathological and behavioral abnormalities associated with epilepsy.

## 1. Introduction

Epilepsy is a chronic neurological disorder characterized by recurrent, spontaneous seizures resulting from abnormal bioelectrical activity in the brain [1]. This prevalent neural pathology affects approximately 50–65 million individuals worldwide [2,3]. One of the most common forms of epilepsy is temporal lobe epilepsy (TLE), in which the epileptogenic focus is located in the temporal lobe [4]. The hallmark of TLE is the degeneration of hippocampal neurons, particularly in the areas of CA1 and CA3 [5].

TLE also results in the manifestation of associated cognitive and psychoemotional disturbances that adversely affect patients’ quality of life [6]. Memory impairments are a common cognitive issue in patients with epilepsy [7], with patients suffering from TLE particularly affected due to epileptogenic foci affecting memory consolidation structures, including the hippocampus [8]. Additionally, epilepsy patients have a higher risk of developing anxiety disorders, personality disorders, psychosis, and attention deficit hyperactivity disorder [9]. Patients with TLE experience challenges in social interactions, specifically in comprehending the mental state of others and identifying emotions [10,11].

Despite continuous research and the availability of anti-seizure medications, approximately 30% of patients with epilepsy do not achieve complete remission [12]. Existing medications can prevent seizures but do not address the underlying mechanisms of epileptogenesis or prevent the development of epilepsy [13,14]. Additionally, many available anti-seizure medications have adverse psychological effects on patients [15,16]. Therefore, it is crucial to discover new treatment methods for epilepsy.

By focusing on the underlying disease mechanisms and primary signaling pathways, it may be possible to prevent or halt the progression of epileptogenesis and provide more effective treatments for epilepsy [14]. Unfortunately, the precise mechanisms of epileptogenesis are still not completely understood, and potential targets for therapy development remain hypothetical [17,18].

Several mechanisms contribute to the development of epilepsy, and neuroinflammation is one of them [19,20,21,22]. Neuroinflammation has been described as a common pathogenic mechanism promoting seizures in animal models of acquired epilepsy and drug-resistant epilepsy in humans. Neuroinflammation involves structural and functional changes in glial and immune cells in the central nervous system, along with dysfunction of the blood–brain barrier (BBB). This imbalance results in heightened production of inflammatory mediators such as IL-1β, IL-6, TNF-α, and IFN-γ [20].

Previous studies, including our own, have demonstrated that anakinra, an interleukin-1 receptor antagonist, reduces epileptogenesis and improves seizure-related outcomes [23,24,25]. Anakinra blocks IL-1 receptor-mediated signaling, which might modulate the inflammatory response and decrease neuronal hyperexcitability. Administering anakinra during the latent phase of the lithium-pilocarpine model significantly reduced the duration and frequency of spontaneous recurrent seizures (SRS) in rats during the chronic phase. Additionally, anakinra prevented certain behavioral impairments, such as motor hyperactivity and disturbances in social interactions, during both the latent and chronic phases. Histological analysis showed that anakinra also reduced neuronal loss in the CA1 and CA3 regions of the hippocampus but did not prevent astro- and microgliosis [23].

Lamotrigine, an anticonvulsant drug, is commonly prescribed for managing epilepsy. Its efficacy is linked to its ability to block voltage-gated sodium channels, which reduces abnormal electrical activity in the brain [26]. More recently, evidence indicates that lamotrigine has antiepileptogenic properties. Lamotrigine pretreatment, administered at doses of 10 and 20 mg/kg, resulted in a significant decrease in seizure stages and generalized seizure durations in the rat pentylenetetrazole kindling model [27]. Additionally, electrophysiological studies illustrated that lamotrigine pretreatment eliminated the heightened population spike amplitude in the hippocampus [27]. The study by Stratton et al. (2003) demonstrated the antiepileptogenic-like effects of lamotrigine in a rat amygdala kindling model [28]. However, a subsequent study by Nissinen et al. (2004) did not show whether lamotrigine had disease-modifying or antiepileptogenic effects [29]. In a rat lithium-pilocarpine model of TLE, Wang et al. (2019) found that lamotrigine decreased the frequency of SRS in a dose-dependent manner and limited neuronal loss as well as astrogliosis in the hippocampus [30].

Since both anakinra and lamotrigine have antiepileptogenic properties but work via different mechanisms, we compared their individual and combined effects. We utilized the lithium-pilocarpine model to reproduce the major phases of epileptogenesis specific to temporal lobe epilepsy. Our assessment involved measuring the occurrence of SRS with different treatment options, as well as neuronal death and behavioral characteristics of the animals.

## 2. Results

### 2.1. The Effect of Treatment with Anakinra, Lamotrigine, and Their Combination Effect on Neurological Parameters

**Figure 1:**
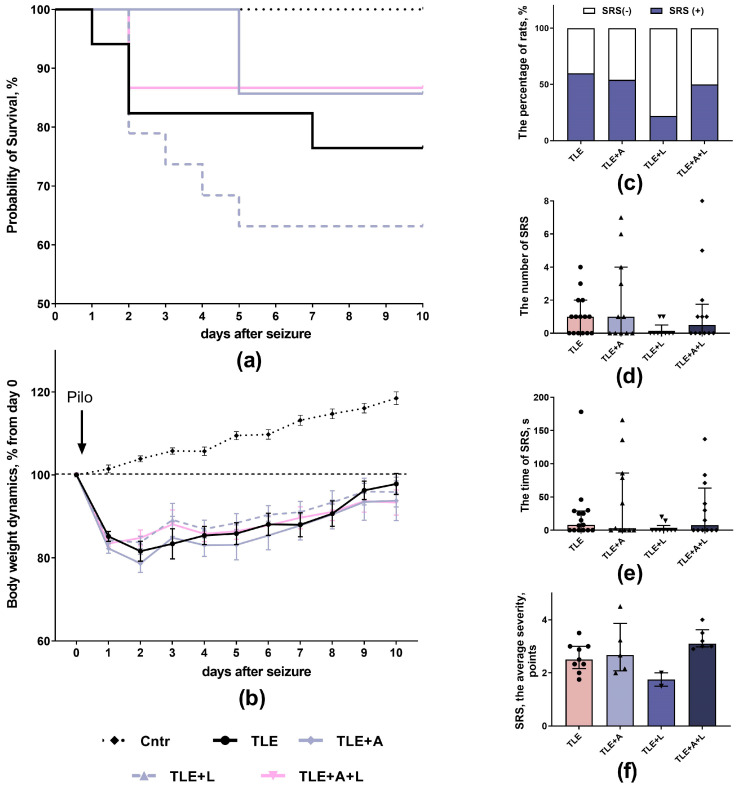
Effect of treatment with anakinra, lamotrigine, and their combination on neurological parameters. (**a**) Kaplan–Meier survival curves. (**b**) Body weight dynamics. (**c**) Percentage of animals showing SRS during the 40 h observation period. (**d**) Number of SRS in the groups during 40 h of observation. (**e**) Duration of SRS episodes in the groups during 40 h of observation. (**f**) Seizure severity according to the Racine scale. Cntr—control rats; TLE—post-SE lithium-pilocarpine untreated group of rats; TLE+A, TLE+L, TLE+A+L—rats treated with anakinra, lamotrigine, and their combination. Each point represents one animal. Data are presented as mean ± standard error of the mean (**b**) or median and interquartile range (**d**–**f**).

The study evaluated body weight changes and survival of rats treated with anakinra and/or lamotrigine for 10 days after pilocarpine administration. The TLE+A+L group receiving combination treatment showed no mortality after two days of treatment. Mortality was observed within the first 5 days in the TLE+A and TLE+L groups and within the first 7 days in the untreated TLE group. However, due to the relatively low mortality across all groups, these differences did not reach statistical significance (Figure 1a; Long Rank test; χ^2^ = 4.27; *p* = 0.23).

After administration of pilocarpine, the body weight of all experimental animals decreased by approximately 15–20% and slowly recovered afterward (Figure 1b). The body weight dynamics of TLE rats differed significantly from controls, according to two-way ANOVA analysis (F_40,620_ = 6.45, *p <* 0.001). However, when excluding control animals, no significant differences were found between treated and untreated TLE animals (F_30,420_ = 0.48, *p =* 0.99).

The severity of spontaneous recurrent seizures (SRS) was evaluated 3.5 months after pilocarpine administration. This was during the chronic phase of the model. Over a period of 40 h, the free behavior of rats was recorded. SRS occurred in 9 out of 15 (60%) untreated TLE animals, 6 out of 11 (54.5%) treated with anakinra, 2 out of 9 (22.2%) rats treated with lamotrigine, and 6 out of 12 (50%) rats receiving the combination treatment (Figure 1c).

The number and duration of seizures did not differ significantly between treated and untreated animals (Figure 1d,e). Analysis of seizure severity based on the Racine scale was only performed in animals with SRS. The study revealed a significant effect of treatment (H_4,22_ = 7.90; *p* = 0.048), but intergroup differences were not statistically significant (Dunn’s post hoc test, *p* > 0.05).

### 2.2. Anakinra, Lamotrigine, or Their Combination Can Prevent Neuronal Death in the Hippocampus following Pilocarpine-Induced Status Epilepticus

**Figure 2:**
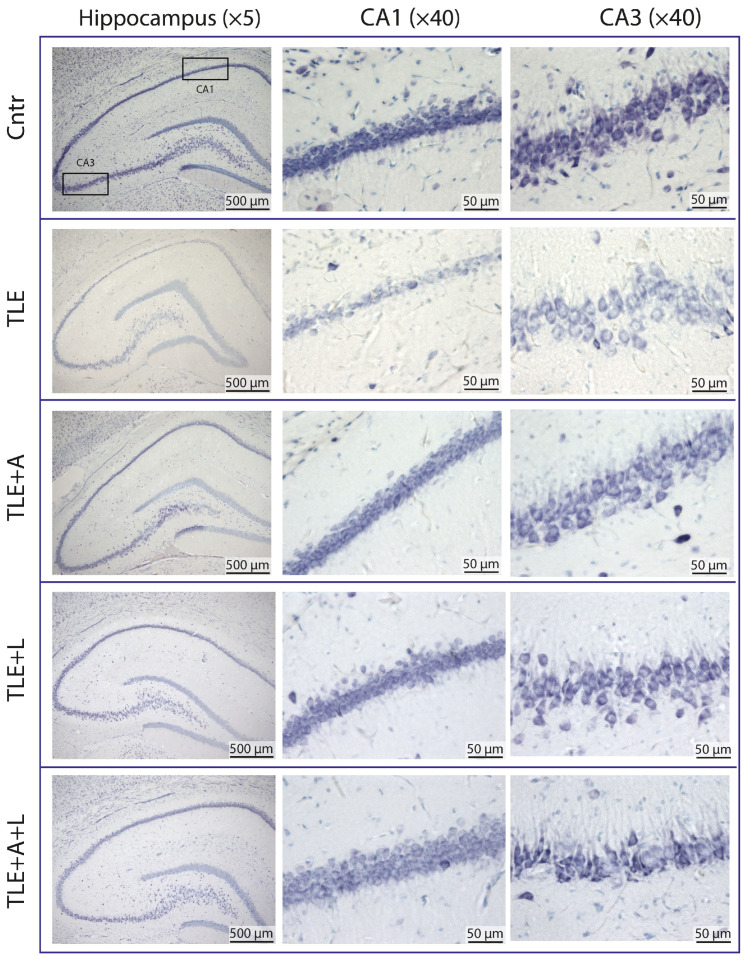
Representative Nissl-stained sections of the hippocampus of control rat (Cntr), post-SE lithium-pilocarpine untreated rat (TLE), and post-SE rats treated with anakinra (TLE+A), lamotrigine (TLE+L), or their combination (TLE+A+L).

A histological study was conducted to assess the effectiveness of administering anakinra, lamotrigine, and its combinations in the lithium-pilocarpine model. Neuron counts in the pyramidal layers of the hippocampal CA1 and CA3 areas were obtained at 120 days post-SE using Nissl-stained brain sections (Figure 2). The counts were taken for the TLE (*n* = 9), matched control (*n* = 6), and after the administration of anakinra (TLE+A, *n* = 6), lamotrigine (TLE+L, *n* = 6), and the combination of these drugs (TLE+A+L, *n* = 9).

**Figure 3:**
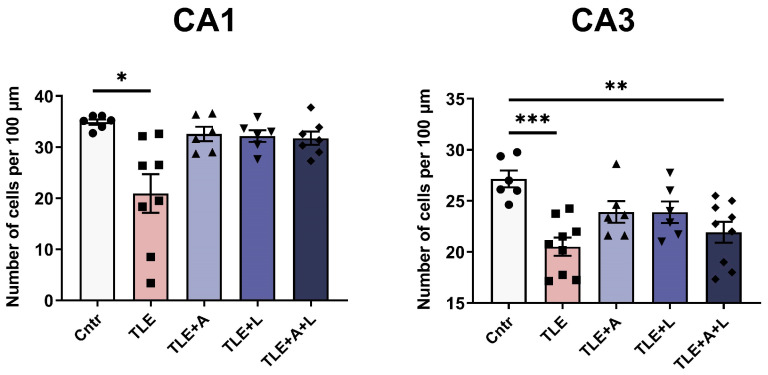
Statistical data on the number of neurons per 100 µm length of cell layer in hippocampal areas CA1 and CA3 of control rats (Cntr), post-SE lithium-pilocarpine untreated rats (TLE), and post-SE rats treated with anakinra (TLE+A), lamotrigine (TLE+L), or their combination (TLE+A+L). Markers indicate individual values per rat. Columns show mean values, and error bars show the standard error of the mean. * *p* < 0.05, ** *p* < 0.01, *** *p* < 0.001 (post hoc Games-Howell test for CA1; post hoc Tukey’s test for CA3). Differences between treated and untreated TLE rats were found only when the ANOVA contrast method was used (for details please refer to the text and Table A1).

In the CA1 region, the variances displayed a significant difference (Levene’s test, *p* = 0.001). Consequently, we applied Welch’s one-way ANOVA, which indicated a substantial impact of status epilepticus induced using pilocarpine on neuronal death in the CA1 hippocampal regions (W_4,13.1_ = 4.59, *p* < 0.001). The Games-Howell test demonstrated notable distinctions in the number of neurons in the hippocampal CA1 field between the control group and the group of animals after pilocarpine-induced seizures (Figure 3). Both administering lamotrigine and anakinra separately and in combination, prevented neuronal death. The ANOVA contrast method indicated a significant difference between treated and untreated TLE rats (*t* = 2.93; *p* = 0.02), but no significant difference was detected between treating with anakinra and lamotrigine separately versus using them in combination (*t* = 0.4; *p* = 0.70).

As there were no significant differences in the variances of cell numbers in the CA3 field between groups (Levene’s test, *p* = 0.75), we utilized one-way ANOVA. This analysis demonstrated significant differences in the number of neurons in the CA3 field of the hippocampus between the control group and the group of animals after pilocarpine-induced SE (F_4,31_ = 6.28, *p* < 0.001). The ANOVA contrast method illustrated a notable difference between the untreated and treated TLE rats (*t* = 2.5; *p* = 0.02). However, in contrast to the CA1 region, a significant reduction in the number of neurons was observed in the CA3 region of the hippocampus, not only in the group of animals that experienced pilocarpine-induced status epilepticus without treatment but also in the group that received a combination of lamotrigine and anakinra after status epilepticus. Tukey’s post hoc test confirmed this finding (Figure 3).

Thus, the use of lamotrigine, anakinra, or their combination was found to be generally effective in preventing neuronal death in the hippocampus after pilocarpine-induced status epilepticus. However, administration of the drugs alone was more effective in preventing neuron loss in the CA3 field of the hippocampus than the combination treatment.

### 2.3. Behavioral Disturbances May Be Improved with Anakinra, Lamotrigine, or Their Combination

We previously demonstrated that epileptogenesis in the lithium-pilocarpine model of temporal lobe epilepsy results in motor activity disorders, altered levels of anxiety, communicative behavior changes, and memory impairments in experimental rats [31]. Therefore, this study aimed to investigate whether the therapy utilized—particularly combination therapy with anakinra and lamotrigine—could prevent TLE rats from developing behavioral disorders.

#### 2.3.1. Treatment Effects on Activity Levels, Exploratory Behavior, and Anxiety in Experimental and Control Animals in the Open Field Test

**Figure 4:**
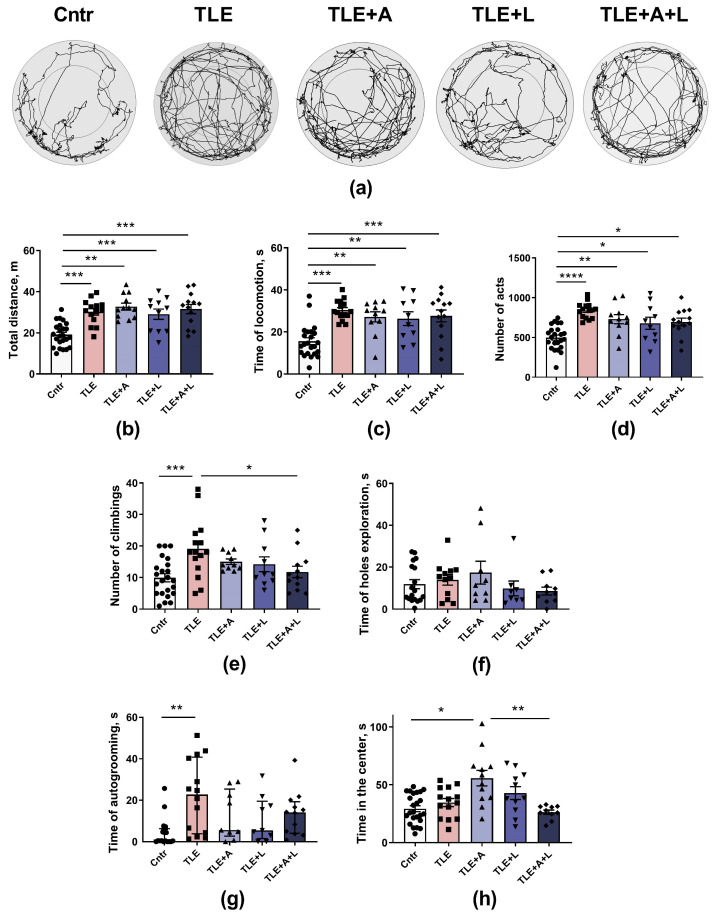
Behavior of rats in the Open field test. (**a**) Representative examples of tracks in the open field of control rat (Cntr), post-SE lithium-pilocarpine untreated rat (TLE), and post-SE rats treated with anakinra (TLE+A), lamotr igine (TLE+L), or their combination (TLE+A+L). Statistical data on total distance traveled by rats in the open field (**b**), time of locomotion (**c**), number of acts (**d**), number of climbs (**e**), time of hole exploration (**f**), time of grooming (**g**), time in the center of the field (**h**). Data are presented as mean and standard error of the mean (**b**–**f**,**h**) or median and interquartile range (g). * *p* < 0.05, ** *p* < 0.01; *** *p* < 0.001,**** *p* < 0.0001, Tukey’s post hoc test (normally distributed data) or Dunn’s multiple comparison test (non-normally distributed data). Each point represents the value of a different animal. More differences between treated and untreated TLE rats were found when the ANOVA contrast method was used (for details, please refer to the text and Table A1).

The locomotor activity of rats in the Open field was analyzed in terms of locomotion time and traveled distance (Figure 4a–c). The TLE animals that did not receive treatment exhibited significantly higher locomotor activity than the control group. Specifically, the total distance increased by approximately 1.5-fold (F_4,67_ = 13.1; *p* < 0.001), and the total locomotion time increased by 2-fold (F_4,67_ =9.5; *p* < 0.001). None of the treatment variants were able to prevent these impairments (Table A1).

To evaluate the rats’ overall activity level, we counted the number of distinct behavioral acts (Figure 4d). This measure significantly increased in both untreated and treated TLE rats (F_4,67_ =10.0; *p* < 0.0001; Tukey’s post hoc test—untreated: *p* < 0.001 and treated *p* < 0.05). The impact of the treatment using anakinra and lamotrigine, alone or combined, was confirmed using the ANOVA contrast analysis (untreated vs. treated *t* = 2.7; *p* = 0.01). No difference was found between combination therapy and monotherapy (*t* = 1.15; *p* = 0.88) (Table A1).

We examined climbing as an indicator of exploratory behavior (Figure 4e). The number of climbs increased in TLE animals (F_4,65_ = 4.49; *p* < 0.01), but the combination treatment prevented this change (Tukey’s post hoc test, *p* < 0.05). ANOVA contrast indicated efficacy of the treatment (*t* = 2.6; *p* = 0.013), but the combined treatment did not show a significant difference from monotherapy use (ANOVA contrast; *t* = 1.15; *p* = 0.25) (Table A1). The time of hole explorations, another indicator of exploratory behavior, did not differ significantly between groups (Figure 4f).

To evaluate the impact of the treatment on animal anxiety, we analyzed the duration of grooming (Figure 4g) and the time spent in the center area of the Open field (Figure 4h). The untreated TLE rats demonstrated an increase in grooming, indicating a higher level of anxiety (H_5,63_ =14; *p* < 0.01; Dunn’s post hoc test *p* < 0.01). This effect was not observed in any of the treated groups. Therefore, treatment decreased the anxiety increase caused by TLE. In addition, when anakinra was administered, a significant increase in time spent in the center of the open field was observed (W_4,28.5_ = 6.0; *p* = 0.001), indicating reduced anxiety levels compared to the untreated animals. The anakinra-treated group showed better results than the combination-treated group (Games-Howell post hoc test *p* < 0.05), while the combination-treated group showed no significant difference from the control group, which was confirmed using ANOVA contrast (*t* = 3.86; *p* = 0.001).

Thus, the treatment alleviated the impairments in activity levels, exploratory behavior, and anxiety. There was no difference in effectiveness between anakinra and lamotrigine monotherapy and the combined treatment for most parameters.

#### 2.3.2. Social Behavior

**Figure 5:**
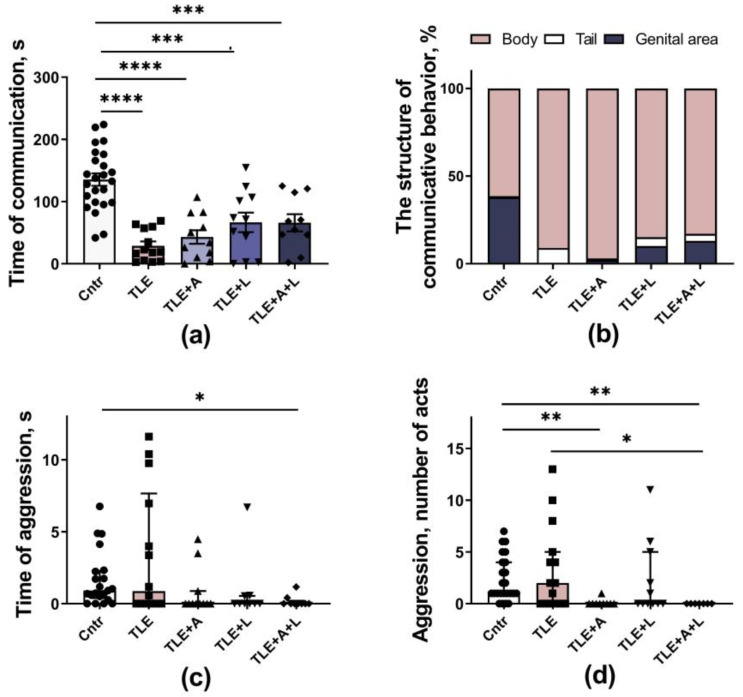
The behavior of the rats in the Social test. (**a**) Time of communication. (**b**) Percentage of time of different types of communicative behaviors. (**c**) Time of aggressive behavior. (**d**) A number of acts of aggressive behavior. * *p* < 0.05, ** *p* < 0.01; *** *p* < 0.001,**** *p* < 0.0001; Tukey’s post hoc test (normally distributed data) or Dunn’s multiple comparison test (non-normally distributed data). Data are presented as mean and standard error of the mean (**a**) or median and interquartile range (**c**,**d**). Each point represents the value of a different animal. More differences between treated and untreated TLE rats were found when the ANOVA contrast method was used (see text and Table A1 for details).

Social behavioral disturbances are one of the most prominent psychiatric disorders in humans with epilepsy [32] and in experimental animals in the lithium-pilocarpine model of this disease [33]. Using the Social test, we showed that the duration of communicative behavior in animals with TLE was reduced about 4–5 times compared to controls (Figure 5a; F_4,64_ = 16.9; *p* < 0.001). These data indicate a severe impairment of social behavior in experimental rats. Although a slight increase in communication time was observed in all treated groups, none of the treatments was able to restore communicative behavior duration to the level of the control group.

In addition to reducing communication time, TLE also altered the structure of social behavior (Figure 5b). In control animals, genital sniffing accounted for 38% of total communication time during contact with a stranger, indicating a confident behavior of the resident. At the same time, sniffing of a stranger’s tail is practically not observed in control rats, which, on the contrary, indicates an insecure behavior of the resident (0.5% of the total communication time). Genital sniffing disappeared almost completely in untreated TLE rats (4%), while tail sniffing increased (up to 8%). In lamotrigine-treated animals, genital sniffing reappeared but did not fully recover (TLE+L group—10.1%; TLE+L+A—13.1%).

Indicators of aggressive behavior—time and number of episodes of aggressive acts were group dependent (Figure 5c,d; H_5,64_ = 13.1; *p* = 0.01 and H_5,66_ = 18.6; *p* = 0.001). However, these changes were not associated with an increase in aggression in untreated TLE rats but with a decrease in aggressive behavior (compared to controls) in the group of rats receiving the combined treatment (Dunn’s post hoc test *p* < 0.01). Thus, the treatment applied had a weak effect on the TLE-induced impairment of social behavior.

#### 2.3.3. The Memory Impairments

##### Fear Conditioning Test

**Figure 6:**
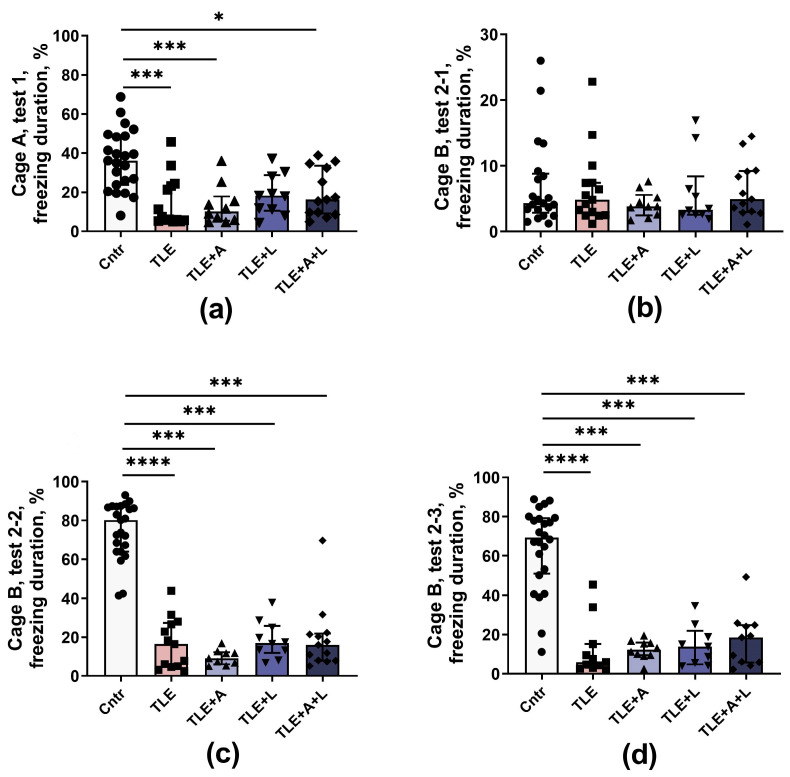
Behavior of rats in the Fear conditioning test. (**a**) Time of freezing in cage A. (**b**) Time of freezing during adaptation to an unfamiliar cage B. (**c**) Time of freezing in the new cage B in response to a tone, a pain-associated stimulus. (**d**) Time of freezing in the novel cage B after cessation of the pain-associated stimulus. * *p* < 0.05, *** *p* < 0.001, **** *p* < 0.0001; Dunn’s multiple comparisons test. The data are presented as the median and interquartile range (**a**,**b**) or as the mean and standard error of the mean (**c**,**d**). More differences between treated and untreated TLE rats were found when the ANOVA contrast method was used (see text and Table A1 for details).

To evaluate fear-related conditioned memory, we utilized the Fear conditioning test (see Figure 6). On the test day, rats were initially placed in cage A, which was associated with shocks and where the conditioning occurred the previous day. Evaluating the total freezing time during this phase enabled us to assess long-term contextual memory. Our findings indicate a significant decrease in freezing time in TLE rats, indicating impaired contextual memory (H_5,70_ = 25.3; *p* < 0.001). The most significant impairments were found in untreated TLE and in the TLE+A groups (Dunn’s post hoc test *p* < 0.01 vs. control). TLE rats treated with lamotrigine exhibited improvement in contextual memory and did not differ significantly from the control group (Figure 6a).

The rats were then transferred to an unfamiliar cage B, where no stimuli were presented for the first 3 min; at this stage, the duration of freezing was short, and no significant differences were observed between groups (Figure 6b). This result indicates that there were no baseline differences between groups in the degree of freezing and that the observed differences were related to memory impairment.

To assess long-term conditioned memory, rats in cage B were presented with a sound cue similar to that used in the learning phase (Figure 6c). Importantly, untreated TLE rats exhibited a significant (about 4-fold) decrease in their freezing response compared to controls. This finding suggests a significant impairment of memory for pain-associated stimuli (H_5,68_ = 45, *p* < 0.001). None of the treatments tested improved these abnormalities.

A high level of freezing was observed in control but not in TLE rats within 1 min after the end of the conditioned stimulus (Figure 6d; H_5,65_ = 39.8, *p* < 0.001), probably indicating a high-stress responsiveness in control animals. The duration of freezing did not differ between the treated and untreated TLE rats in this subtest.

##### Morris Water Maze

**Figure 7:**
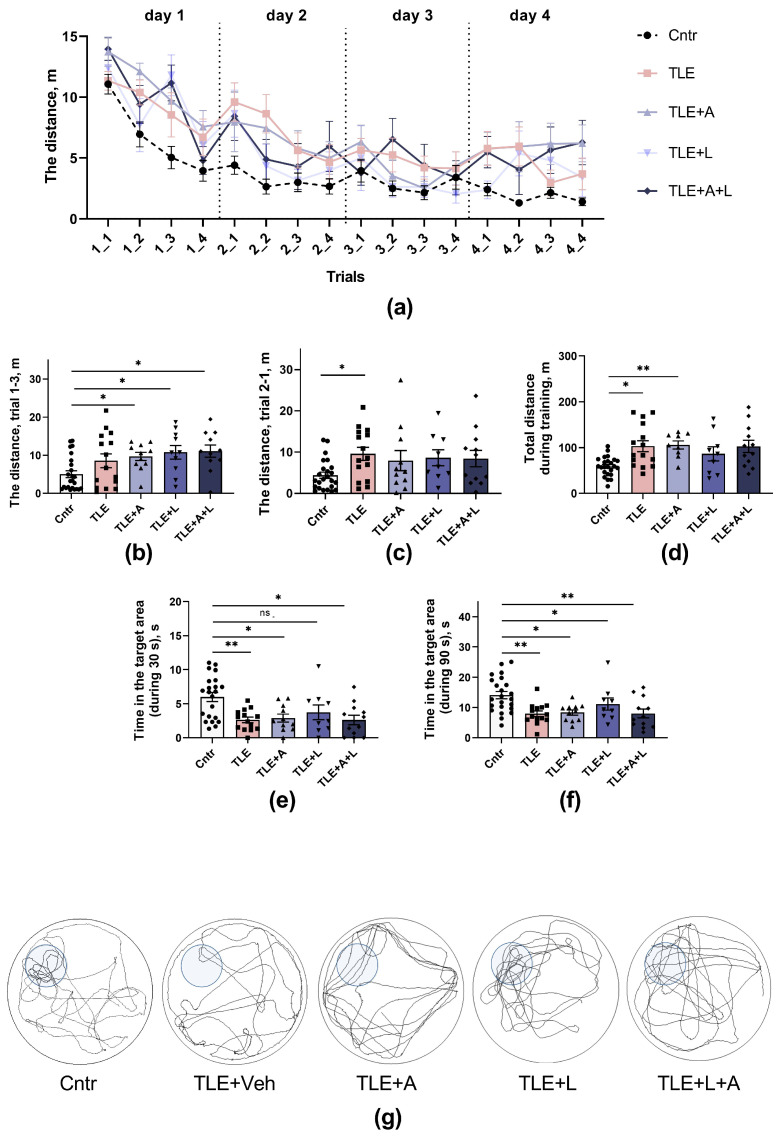
Spatial learning and memory of rats in the Morris water maze. (**a**) Training dynamics (distance traveled to find the platform) over four training days. (**b**) Distance traveled before finding the platform in the third trial of the first training day. (**c**) Distance traveled before finding the platform on the first attempt of the second training day. (**d**) Total distance traveled before finding the platform on all trials for four training days. (**e**–**g**) Long-term spatial memory test on the fifth experimental day, time spent in the target area where the platform was previously located: (**e**) first 30 s; (**f**) for the entire test (90 s). (**g**) Examples of tracks during platform retrieval on test day. Data are shown for the entire test (90 s). The area where the platform was previously located is highlighted. * *p* < 0.05; ** *p* < 0.01, ns—nonsignificant difference, Tukey’s or Games-Howell’s post hoc test. Data are presented as the mean and standard error of the mean. Each point represents the value of a different animal. More differences between treated and untreated TLE rats were found when the ANOVA contrast method was used (see text and Table A1 for details).

Long-term spatial memory was evaluated in the Morris water maze (Figure 7). Rats underwent four-day training sessions (with four trials daily) to locate a submerged platform. Improvement in skills was determined by a decrease in the distance traveled before reaching the platform. The training progress of the rats in the Morris water maze is depicted in Figure 7a. As training progressed, all groups showed an improvement in their ability to find the platform. However, the efficiency of the training varied between groups (mixed ANOVA: trials—F_15,975_ = 24.8, *p* < 0.001; groups—F_4,65_ = 5.34, *p* = 0.001; interaction—F_60,975_ = 1.21, *p* = 0.14).

Control rats learned better than TLE rats. Both short-term and long-term memory impairments were observed in TLE rats. For example, in the third trial of the first day of training (short-term memory), treated TLE rats swam a longer distance to the platform compared to controls (Figure 7b, one-way ANOVA; W_4,27.3_ = 5.3; *p* < 0.01), a similar result was observed in untreated TLE rats. In the first test on the second day, the worst results were found in untreated TLE animals (Figure 7c), but treated rats had similar results (one-way ANOVA; F_4,65_ = 2.8; *p* = 0.03). Animals from different groups differed in the total distance traveled in all sixteen trials (Figure 7d; one-way ANOVA; W_4,24.8_ = 8.6; *p* < 0.001), with the distance traveled being maximal in the TLE and TLE+A groups.

Overall, the analysis of learning showed that TLE leads to impaired learning in the Morris water maze, with no significant differences between treated and untreated rats (ANOVA contrast results are given in Table A1).

To test long-term spatial memory (Figure 7e–g), the platform was removed on the fifth experimental day, and the animals were placed in the pool for 90 s. Staying in the target area where the platform was previously located for a long period of time was considered a high spatial memory performance. Because most control rats would first search for the platform in a given area and then, if they did not find it, swim away to search elsewhere, we examined the time spent in the target area not only during the entire test (90 s, Figure 7f) but also during the initial period (30 s, Figure 7e). In both cases, the groups differed significantly in the amount of time spent in the target area: 30 s—W_4,26.9_ = 4.8; *p* < 0.01; 90 s—F_4,64_ = 5.36; *p* < 0.001. Maximum memory impairment was observed in untreated TLE rats, but treated rats were also significantly different from controls (Tukey’s post hoc test, *p* < 0.05). Lamotrigine monotherapy reversed these abnormalities when the first 30 s of the search were analyzed (Figure 7e, differences from control *p* > 0.05). However, the ANOVA contrast method revealed no significant differences between animals receiving different treatments (Table A1).

Thus, TLE rats showed a decrease in spatial memory. Treatment with lamotrigine reduced memory abnormalities but did not completely prevent them.

## 3. Discussion

In the present study, the rat lithium-pilocarpine TLE model [34] was utilized to examine the effects of anakinra and lamotrigine alone and in combination on epileptogenesis. Administration of these drugs during the initial 10-day period following pilocarpine-induced status epilepticus lessened the severity of SRS. In addition, anakinra and lamotrigine alone and in combination significantly ameliorated a number of behavioral deficits and reduced, but did not completely abolish hippocampal neuronal loss. The effectiveness of the combined treatment did not significantly vary from that of anakinra and lamotrigine monotherapy. Thus, it can be inferred that both anakinra and lamotrigine have a disease-modifying effect in this TLE model.

Since acquired epilepsy often appears to be pharmacoresistant, prevention of epileptogenesis is an important goal [35]. Epileptogenesis is the complex process by which a healthy brain becomes epileptic. Several animal models are used to study epileptogenesis and provide valuable insights into the underlying mechanisms of epilepsy [36]. These models allow researchers to study various aspects of epileptogenesis, including seizure development, neuronal loss, synaptic reorganization, neuroinflammation, and metabolic changes in the brain. Some commonly used animal models to study epileptogenesis include (1) kindling models, in which epileptic activity is induced by repeated application of low-dose convulsant drugs or electrical stimulation of specific brain regions, such as the amygdala [37]. (2) Post-status epilepticus (post-SE) models in which high doses of a convulsant agent such as kainate or pilocarpine are injected systemically [37]; (3) brain injury models, in which epilepsy develops after brain damage or stroke [38,39]. Compared to kindling models, the post-SE lithium-pilocarpine model is deemed more reliable in identifying drugs with antiepileptogenic properties. For instance, in models of kindling, drugs are usually given before the daily kindling sessions [27,28], making it impossible to rule out the possibility that the anticonvulsant impact of the drugs mitigates kindling-dependent epileptogenesis [30].

Evidence of antiepileptogenic efficacy is increasing for numerous compounds. In a recent review, the authors listed 156 compounds with published reports of antiepileptogenic efficacy [40]. In this study, we tested the antiepileptogenic efficacy of lamotrigine and anakinra. Lamotrigine is a conventional antiepileptic medication utilized to treat both focal and generalized epilepsy [41]. The pharmacological effect of lamotrigine involves the blockage of potential-dependent sodium [42] and N- and P/Q-type calcium channels on presynaptic nerve terminals [43,44]. Lamotrigine prevents excessive release of glutamate, protecting nerve cells from glutamate-induced neurotoxicity [45,46]. After status epilepticus induced using pilocarpine, neuronal networks become more excitable with pathologically high background activation of the glutamatergic system [47,48]. To minimize this effect, we employed lamotrigine in our study. Previously, various experimental models of epilepsy, including the lithium-pilocarpine model of temporal lobe epilepsy, have shown the neuroprotective and anti-epileptogenic effects of lamotrigine [30,49].

Another factor in favor of choosing lamotrigine was its ability to positively impact the mental state of patients, setting it apart from numerous other antiepileptic medications that can trigger psychoemotional and cognitive dysfunctions. Research has demonstrated that lamotrigine displays a strong antidepressant impact, making it suitable for mood stabilization in individuals with bipolar disorder [50]. It should also be noted that lamotrigine has demonstrated the potential to inhibit the release of proinflammatory cytokines in models of neuroinflammation [51].

Anakinra, a recombinant interleukin-1 receptor antagonist, has shown potential in the treatment of some types of epilepsy, particularly in febrile infection-related epilepsy syndrome (FIRES) [24,52] and as an antiepileptogenic drug [23]. The mechanisms by which anakinra exerts its effects in epilepsy are not fully understood. However, it is believed to change the expression of various genes, modulate the immune response, and reduce inflammation [23,53,54]. Neuroinflammation is associated with increased production of proinflammatory cytokines such as IL-β, IL-6, TNF-α, and others. Increased expression of proinflammatory genes, particularly IL-1b and TNF-α, was found in the hippocampus and anterior temporal cortex of patients with TLE and hippocampal sclerosis [55,56]. High levels of IL-1β enhance excitation in the CNS by increasing the release of excitatory transmitters such as glutamate or ATP [57]. IL-1β enhances NMDA-mediated Ca^2+^ influx into the cell [58], which may induce the characteristic hippocampal neuronal death seen in epilepsy [59]. In addition, IL-1β may increase neuronal excitability by down-modulating the astrocytic glutamate transporter (GLT-1) [60], which results in impaired glutamate clearance that has been identified as one of the causative factors in drug-resistant epilepsy [61]. Increased levels of proinflammatory cytokines, including IL-1, may not only be associated with increased excitability but may also be responsible for the development of behavioral abnormalities characteristic of epilepsy [62]. We and other researchers have previously demonstrated that IL-1 receptor blockade therapy decreases seizure development and neurodegenerative changes in the brain, as well as reducing the severity of comorbid behavioral disorders. However, it does not completely prevent them [23,63,64].

One of the key elements in epileptogenesis is neuronal death, which has been widely studied in the context of acquired epileptogenesis [65]. Traditionally, it has been proposed that neuronal death is necessary for epileptogenesis, as the loss of synaptic input from dying neurons is considered a critical signal to induce axonal sprouting and rewiring [65]. However, recent studies have challenged this concept and suggested that neuronal death may not be essential for epileptogenesis, particularly in the immature brain [65]. Our study demonstrates that both lamotrigine and anakinra provide neuroprotective effects and reduce neuronal death in the hippocampus, but they do not fully prevent spontaneous recurrent seizures. Thus, our data are consistent with the results of previous studies, which also showed that preventing neuronal loss is an important but not always sufficient factor to prevent epileptogenesis [66,67,68]. It should also be noted that both anakinra and lamotrigine showed a more pronounced neuroprotective effect in the CA1 hippocampal region than in CA3. The degree of neuronal damage in different hippocampal regions may differ depending on the age of the animals and the model used [69,70,71,72]. Additionally, the neuroprotective effects of anakinra and lamotrigine may be different for these hippocampal areas. For example, it has been previously shown that pyramidal cells in the CA3 area are highly excitable due to the recurrently connected network via axonal collaterals within the CA3 area, and this could lead to increased neuronal activity and potentially stronger neurodegeneration during epileptic seizures [73,74]. Therefore, it can be hypothesized that the treatment effect would be more pronounced for the CA1 region of the hippocampus than for the CA3 region.

In our study, we also aimed to investigate the effect of drugs on behavioral disturbances in rats. This was motivated by the observation that a comorbid diagnosis of epilepsy and psychiatric disorders was found to predict pharmacoresistance [75]. Analogous findings have also been reported in a rat model of epilepsy [76]. Another point for this study is that epilepsy patients also have a higher risk of developing anxiety disorders, psychosis, attention deficit hyperactivity disorder, and various personality disorders [9]. Patients with TLE have difficulties in social interaction, especially in modeling the mental state of others and recognizing emotions [10,11]. In addition, patients with TLE and hippocampal sclerosis show impairments in several types of memory [8,77] and social behavior [78], which may also be related to hippocampal dysfunction [79].

Similar behavioral abnormalities are observed in animal models of temporal lobe epilepsy. Pilocarpine and lithium-pilocarpine rodent models of TLE are characterized by hyperactivity, impaired memory, social behavior, and anxiety [33,80,81], which was confirmed in the present study. Behavioral abnormalities often manifest in the latent period of the model when SRS is barely observed [82].

We have shown that treatment with anakinra, lamotrigine, or their combination in the early stages of epileptogenesis ameliorated impairments in motor activity, exploratory behavior, and anxiety in the open field, reduced aggression in the social test but only slightly improved TLE-induced impairments in communicative behavior and memory. At the same time, the efficacy of the combined treatment was almost identical to that of anakinra and lamotrigine monotherapy.

We have previously shown that administration of anakinra for 10 days after pilocarpine-induced status epilepticus attenuates, but does not completely prevent, some behavioral deficits that develop during the latent and chronic phases of the lithium-pilocarpine model of TLE [23]. Mazarati et al. [62] found that intrahippocampal administration of interleukin-1 receptor antagonist, an analog of anakinra, ameliorated psychoemotional disturbances in the lithium-pilocarpine model of TLE in rats. The present study mainly confirms the previously obtained data.

Our data obtained during treatment with lamotrigine are consistent with clinical observations showing that lamotrigine also has a positive effect on the mental state of patients with epilepsy. For example, Miller et al. found that lamotrigine reduced depressive symptoms in patients with epilepsy [83], and Kato et al. showed a decrease in aggression in patients with TLE after treatment with lamotrigine [84]. In a study performed in rats in the lithium-pilocarpine model of TLE, Mahfoz et al. found that administration of lamotrigine attenuated pilocarpine-induced spatial memory impairments in the Morris water maze [85]. However, in that study, lamotrigine was administered prior to the pilocarpine injection, which may account for a more pronounced effect than we found.

Overall, our study suggests that treatment with anakinra and lamotrigine has a protective effect on the development of some neurodegenerative and behavioral abnormalities during epileptogenesis. Although our study did not confirm the hypothesis that combined therapy is more effective than monotherapy with these drugs, the results suggest that both anakinra and lamotrigine, either alone or in combination, may have clinical value in preventing epileptogenesis.

## 4. Materials and Methods

### 4.1. Animals

This study used male Wistar rats that were 8 weeks old and housed in standard cages with 5–6 rats per cage. The rats had unlimited access to water and food and were exposed to a 12 h cycle (8 p.m. to 8 a.m. light and 8 a.m. to 8 p.m. dark). Rats from various litters were mixed to prevent potential genetic influence and then allocated to either the control or experimental groups. All animal experiments were conducted in accordance with the regulations and standards set forth by the Animal Care and Use Committee of the Sechenov Institute of Evolutionary Physiology and Biochemistry of the RAS. These guidelines adhere fully to the EU Directive 2010/63/EU for animal experiments.

### 4.2. The Lithium-Pilocarpine Model and Treatment

**Figure 8:**
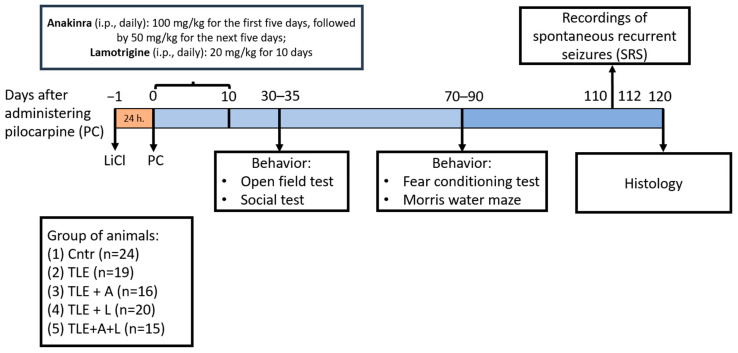
The experimental design.

The experimental plan is shown in Figure 8. Rats received an intraperitoneal (i.p.) injection of 127 mg/kg LiCl. After 24 h, they were given the muscarinic receptor agonist pilocarpine (i.p.). To avoid any peripheral effects of pilocarpine, (−)-scopolamine methyl bromide (1 mg/kg, i.p.) was given 1 h before the pilocarpine injection. Rats were injected with 10 mg/kg of pilocarpine every 30 min until they experienced seizures with a score of 4 on the Racine scale [86]. Rats that did not have seizures after the fourth injection (totaling 40 mg/kg of pilocarpine) were excluded from the study. After 75 min, reaching stage 4 seizures, diazepam (10 mg/kg, i.p.) was given to stop the convulsions.

A total of 94 rats were included in the study. The control group of rats (Cntr; *n* = 24) received LiCl and saline without pilocarpine injection. The experimental rats, injected with pilocarpine, were randomly allocated into one of four groups based on the type of treatment. One group remained untreated (TLE; *n* = 19), while the remaining three groups received the following treatments: (1) TLE+A group (*n* = 16), which was treated with anakinra on the following schedule: 5 injections of 100 mg/kg, i.p. once a day, then five injections of 50 mg/kg once a day; (2) TLE+L group (*n* = 20) was treated with lamotrigine dissolved in DMSO, 20 mg/kg, i.p. once a day; (3) TLE+A+L group (*n* = 15) received a combination of lamotrigine and anakinra in the doses described above. The first injection of all drugs was given one hour after the cessation of seizures. Effective doses and drug delivery regimens have been established in previous studies [23,30]. 

### 4.3. Survival Rate and Body Weight

Survival rate and body weight were monitored for ten days following pilocarpine-induced status epilepticus. The animals were initially given moist food and received subcutaneous injections of 5% glucose (2–3 mL) to increase their survival.

### 4.4. Spontaneous Recurrent Seizures (SRS)

110–112 days after receiving pilocarpine injections, the presence of SRS was assessed. Each rat was placed in a clear cage with unrestricted access to food and water, and their free behavior was videotaped for 40 h. The collected data were analyzed to determine the total and average duration and frequency of SRS.

### 4.5. Behavioral Testing

Behavioral assessment was conducted in the chronic phase of the model. If seizures occurred in the rats during testing, testing was halted, and the results of that test were not taken into account. Experiments were performed between 6 p.m. Furthermore, 11 p.m. Video recordings of all tests were analyzed with “Tracking”, Version 3.2 and “Field 4”, Version 4 software (Institute of Experimental Medicine, St. Petersburg, Russia). The behavioral tests were conducted by an experienced researcher who was blinded to a group of animals.

#### 4.5.1. Open Field Test

The Open field test was used to assess motor and exploratory activity. Each rat was placed in the center of a circular arena 1 m in diameter with 30 cm high walls. The arena had 4 cm diameter holes in the floor and was illuminated with a luminance of 8 Lx. The movements of each rat were monitored and recorded for 5 min. Several parameters were calculated to assess locomotor activity, including total distance traveled, time spent moving, and immobility. To assess the anxiety level of the rats, the time spent in the center of the arena (which is one-third of the arena diameter) was measured. In addition, the frequency and duration of specific behaviors, such as climbing, rearing, sniffing, and exploring holes (an indicator of exploratory activity), as well as auto grooming and freezing (indicators of anxiety), were determined. The arena was cleaned between trials to minimize the influence of olfactory cues.

#### 4.5.2. Fear Conditioning Test

**Figure 9:**
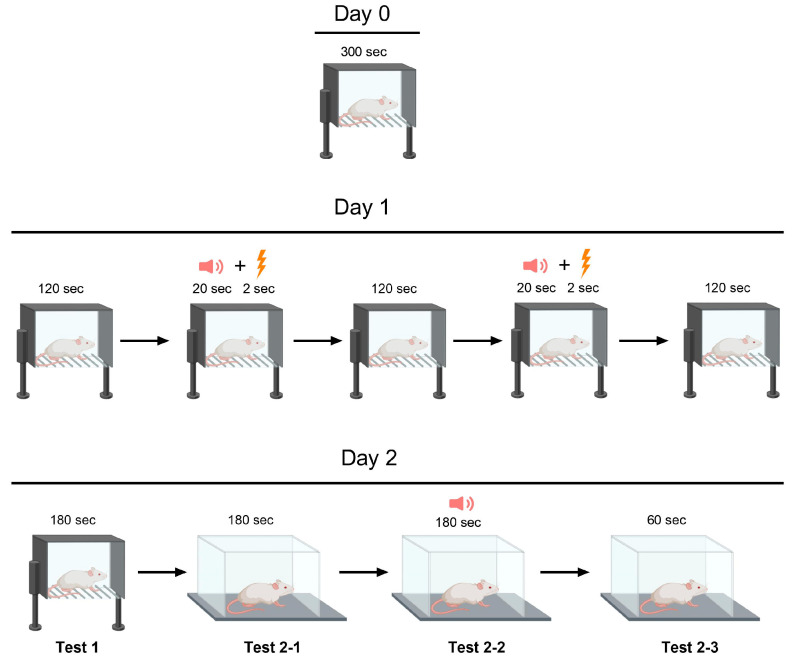
Fear Conditioning Test Scheme.

Two Plexiglas cages were used in this experiment (Figure 9). Cage A measured 45 × 30 cm with a height of 20 cm and had a conductive floor. Cage B was larger at 60 × 30 cm with a height of 40 cm and did not have a conductive floor. On Day 0, the rats were habituated to Cage A for five minutes. On day one, each rat was placed in cage A to undergo the conditioning stages. The habituation period lasted for two minutes, followed by the presentation of an 80-dB sound for 20 s as an auditory cue. Immediately following the auditory cue, a light foot shock of 0.6 mA lasting 2 s was administered as an aversive stimulus. After a two-minute break, the previous step was repeated. During the final two min of the test on Day 1, no sound was produced. On the second day, the rat was returned to Cage A for a 3 min period without any cues or stimulation to evaluate its contextual fear memory (contextual conditioning test, test 1). The animal was then shifted to a new environment (Cage B) featuring dissimilar olfactory and visual cues (vanillin drops on the floor and geometrical figures on the walls). After 3 min of habituation (test 2-1), an 80-dB sound was emitted for 3 min to measure the rat’s fear response to a current-associated sound stimulus (cued conditioning testing, test 2-2). During the final minute of the experiment, no stimuli were presented to the rat, while its response to the conditioned stimulus or the cage was recorded as the time spent freezing (test 2-3). Fear-related memory was analyzed via the percentage of time spent freezing in relation to the duration of each step, which varied in length. Day 1’s immediate post-foot-shock freezing response reflected short-term fear-associated memory, while Day 2’s freezing response reflected long-term fear-associated memory.

#### 4.5.3. Social Interaction Test

To assess social behavior, a social interaction test was conducted. First, a resident rat was placed in a 60 × 30 cm Plexiglas cage with a height of 40 cm for 30 min to acclimate to the new environment prior to the test. Then, an adult male intruder weighing at least 10 g less than the resident rat was placed in the same cage for 5 min. Behaviors exhibited during this interaction were quantified, including aggression, communication (such as grooming the intruder’s body and sniffing the intruder’s tail and genitals), and defense. The total and average time spent performing each behavior was analyzed.

#### 4.5.4. Morris Water Maze

A Morris water maze was used to assess spatial memory and learning ability. A circular pool 150 cm in diameter with walls 70 cm high was filled with water laced with milk (to reduce visibility) and maintained at a temperature of 23 ± 1 °C. Four cues were placed on the walls to serve as spatial reference points: a circle, a square, a triangle, and a cross. The rats had to find a hidden platform (diameter 10 cm) located 1 cm below the water surface and 15 cm from the pool wall. Training was performed for four consecutive days, four trials per day for each rat, with a 5-min break between trials. The starting point for each trial was randomly rotated among the four locations (north, east, south, or west) so that all 4 locations were used during each training day. The platform location remained unchanged. The rat was left on the platform for 30 s after detection and then removed from the pool. An attempt was considered unsuccessful if the rat did not find the platform within 90 s. On test day 5, the platform was removed, and the rat was placed in the pool for 90 s. Time spent in the target area (four times the diameter of the platform) where the platform was previously located was scored.

### 4.6. Histology

Rats were anesthetized with isoflurane, then decapitated, and the brain removed. The brain was then fixed in 4% paraformaldehyde solution for 3–7 days at 4 °C, cryoprotected in 30% sucrose, and stored at −80 °C. Frontal serial sections of 20 µm thickness (−2.76 to −3.6 mm to bregma) were cut on a Bright OTF5000 cryostat (Bright Instrument Co., Ltd., Huntingdon, UK).

Nissl staining was performed with 0.05% thionine solution as previously described [87]. Sections were analyzed on a Leica AF 7000 microscope (Leica Microsystems, Wetzlar, Germany) at ×400 magnification. For morphological analysis, neurons were counted on every 10th slice (a total of 8–10 slices from one rat hippocampus). The distance between analyzed slices was 200 μm. The number of neurons in the digital micrographs was counted in ImageJ 1.52a software (U.S. National Institutes of Health, Bethesda, MD, USA) using the multi-point tool at 100 μm for the cell layer in two hippocampal regions: CA1 and CA3.

### 4.7. Statistical Analysis

Statistical analysis was conducted using IBM SPSS Statistics 20 (IBM, Armonk, NY, USA) and Graph Pad Prism 8 (Graph Pad Software, San Diego, CA, USA) software. Outliers were identified using Dixon’s Q-test. The Kolmogorov–Smirnov test was used to test for normality of distribution. The Kruskal–Wallis H-test with Dunn’s post hoc test was used to evaluate treatment effects for non-normally distributed data in pairwise comparison of groups. In the case of a normal distribution, we performed the Levene test to check for equal variances between groups. If the variances were not significantly different, we used one-way ANOVA and Tukey’s post hoc test. If the variances were significantly different, we used a variant of one-way ANOVA, Welch’s test, and the Games-Howell test for post hoc analysis. The hypothesis of a difference between treated and untreated rats on the studied parameters was tested using the ANOVA contrast method [88,89]. To do this, four contrasts were examined: (1) untreated TLE (−3) vs. all treated animals (TLE+A; +1; TLE+L; +1; TLE+A+L; +1); (2) Monotherapy with anakinra (+1) and lamotrigine (+1) vs. combined therapy (−2). (3) Untreated TLE (−2) and treated with anakinra (TLE+A; +1; TLE+A+L; +1); (4) Untreated TLE (−2) and treated with lamotrigine (TLE+L; +1; TLE+A+L; +1). Contrasts were calculated for normally distributed data with significant values from a one-factor ANOVA.

Weight dynamics after pilocarpine administration, as well as the dynamics of successful rat learning in the Morris water maze, were analyzed using two-way mixed ANOVA. Survival analysis was performed using the log-rank Mantel-Cox test. Fisher’s exact test was utilized to compare the proportions of rats with and without SRS in different groups. Differences were considered significant at *p* ≤ 0.05. Data is presented as mean ± standard error (for normal distribution) or median and interquartile range (for non-normal distribution).

## Data Availability

The data presented in this study are available on request from the corresponding author.

## Appendix A

**Table A1:** Results of ANOVA contrast *.

The Indicators	Untreated TLE Rats (−3) vs. Treated with Anakinra (TLE+A; +1); Lamotrigine (TLE+L; +1) and Combined Treatment (TLE+A+L; +1)	Untreated TLE Rats (−2) vs. Treated with Anakinra (TLE+A; +1) and Combined Treatment (TLE+A+L; +1)	Untreated TLE Rats (−2) vs. Treated with Lamotrigine (TLE+L; +1) and Combined Treatment (TLE+A+L; +1)	Monotherapy with Anakinra (−1) and Lamotrigine (−1) vs. Combined Therapy (TLE+A+L; +2)
**Histological studies**				
Neuronal death in CA1 hippocampal regions	*t* = 2.93; *p* = 0.02	*t* = 2.89; *p* = 0.02	*t* = 2.85; *p* = 0.022	*t* = 0.4; *p* = 0.70
Neuronal death in CA3 hippocampal regions	*t* = 2.50; *p* = 0.022	*t* = 2.08; *p* > 0.05	*t* = 1.56; *p* >0.05	*t* = 1.56; *p* = 0.14
**The Open Field test**				
Time of locomotion	*t* = 1.30; *p* = 0.20	*t* = 1.10; *p* = 0.28	*t* = 1.24; *p* = 0.22	*t* = 0.30; *p* = 0.76
Number of climbing	*t* = 2.55; *p* = 0.013	*t* = 2.50; *p* = 0.015	*t* = 2.68; *p* = 0.009	*t* = 1.15; *p* = 0.25
Number of acts	*t* = 2.66; *p* = 0.01	*t* = 2.31; *p* = 0.02	*t* = 2.74; *p* = 0.98	*t* = 1.15; *p* = 0.88
Time in the center	*t* = 1.40; *p* = 0.17	*t* = 1.20; *p* = 0.24	*t* = 0.24; *p* = 0.01	*t* = 3.86; *p* = 0.001
**Social test**				
Time of communication	*t* = 0.67; *p* = 0.50	*t* = 0.37; *p* = 0.71	*t* = 1.09; *p* = 0.28	*t* = 0.60; *p* = 0.55
**Morris water maze**				
Total distance during training	*t* = 0.25; *p* = 0.80	*t* = 0.22; *p* = 0.83	*t* = 0,56; *p* = 0.58	*t* = 0.26; *p* = 0.80
Time in the target area (during 90 s)	*t* = 0.085; *p* = 0.93	*t* = 0.17; *p* = 0.87	*t* = 0,19; *p* = 0.87	*t* = 0.19; *p* = 0.85
Time in the target area (during 30 s)	*t* = 0.33; *p* = 0.74	*t* = 0.18; *p* = 0.86	*t* = 0,39; *p* = 0.70	*t* = 0.78; *p* = 0.45

* Contrasts were calculated for normally distributed data with significant values of one-factor ANOVA.
